# Review of Health Monitoring Techniques for Capacitors Used in Power Electronics Converters

**DOI:** 10.3390/s20133740

**Published:** 2020-07-03

**Authors:** Hoang-Long Dang, Sangshin Kwak

**Affiliations:** School of Electrical and Electronics Engineering, Chung-Ang University, Heukseok-dong, Dongjak-gu, Seoul 06974, Korea; danghoanglong2692@gmail.com

**Keywords:** capacitance monitoring, capacitance estimation, condition monitoring, capacitor health condition, reliability

## Abstract

Capacitors are critical components of power converter systems as they influence the cost, size, performance, and scale of such systems. However, capacitors exhibit the highest degeneration and breakdown rates among all power converter components due to their wear-out failures and short lifespans. Therefore, condition monitoring is a vital process to estimate the health status of capacitors and to provide predictive maintenance for ensuring stability in the operation of power converter systems. The equivalent series resistance (*ESR*) and the capacitance of the capacitor are two widely used parameters for evaluating the health status of capacitors. Unlike the *ESR*, the capacitance of a capacitor is suitable for the health monitoring of various types of capacitors; therefore, it is more preferable for large-scale systems. This paper presents an overview of previous research addressing this aspect of capacitors and provides a better understanding of the capacitance monitoring of capacitors utilized in power converter systems.

## 1. Introduction

**Figure 1:**
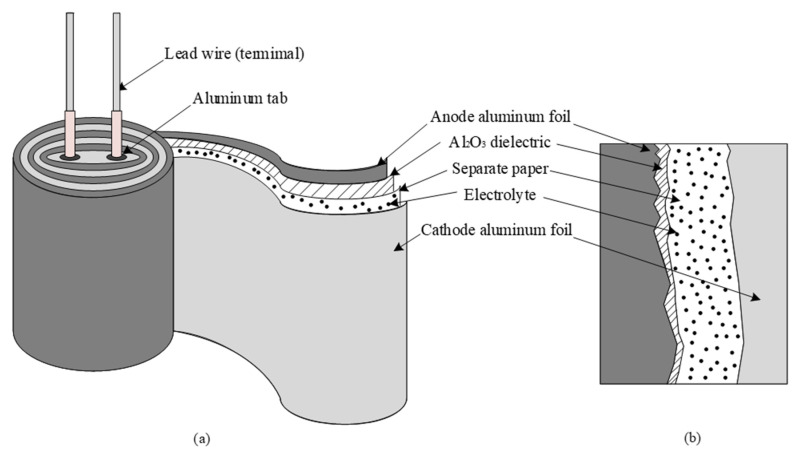
Constructions of an aluminum electrolytic capacitor (Al-Cap): (**a**) cylinder construction; (**b**) cross section of cylinder.

Power electronic converters are employed as interfaces between sources (such as grids, wind turbines, and solar photovoltaic (PVs)) and loads (such as transmission systems, motor drives, electric vehicles, and residential houses). Due to the increased use of power electronic converters for interfacing in power systems, it is essential to maintain the reliability of power converters in order to ensure the stability of system operations. Among the components used in these power converters, capacitors exhibit the highest degeneration and breakdown rates due to their wear-out failures and short lifespans [1]. There are three types of capacitors that are widely used in power electronic systems: aluminum electrolytic capacitors (Al-Caps), metallized polypropylene film capacitors (MPPF-Caps), and high capacitance multilayer ceramic capacitors (MLC-Caps). A major factor that limits the performance of capacitor is the properties of dielectric materials—Al_2_O_3_, polypropylene, and ceramics—that are used in Al-Caps, MPPF-Caps, and MLC-Caps, respectively [2]. The other factors such as design structure, production process, and foil thickness are also important and significantly contribute to the performance of capacitors. Figure 1 illustrates the construction of a cylindrical Al-Cap and its cross section. The Al-Cap consists of two aluminum foils that are parallel plates; the positive foil acts as the anode, whereas the negative foil acts as the cathode. A thin layer of aluminum oxide (Al_2_O_3_) is electrochemically formed (anodic oxidation) on the aluminum surface. This layer serves as the dielectric layer. A spacing paper is used to separate the foils in order to avoid contact between the anode and cathode; thereafter, the foils and the spacing paper are wound together. The foil surface has been electrically etched to increase the effective surface area. The electrolyte is an electrical conductor and serves as a contact to the aluminum foil covered by the aluminum oxide dielectric layer. Aluminum tabs are stitched or cold welded onto the anode and cathode foils in order to connect the capacitor with the outer circuit [3].

**Figure 2:**
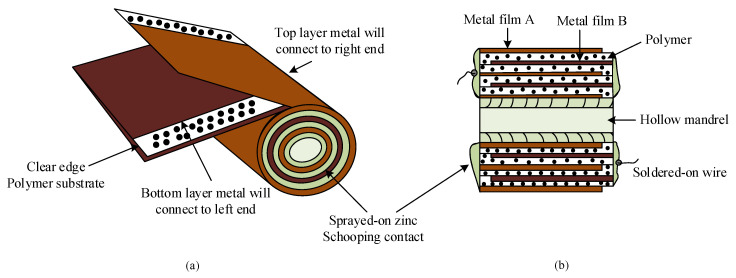
Construction of a metallized polypropylene film capacitor (MPPF-Cap): (**a**) cylinder construction; (**b**) cross section of the cylinder.

Figure 2 presents the typical construction and cross section of a cylindrical MPPF-Cap. The MPPF-Cap contains two pieces of polypropylene film, which are wound into a cylindrical shape over an insulating mandrel. Each strip features a significantly thin metallization layer, typically composed of aluminum, that is applied to one or both sides to serve as the electrodes. The capacitor is wound such that there are the clear margins at each end of the cylinder. A key advantage of this construction is that direct contact between the electrodes and both ends of the cylinder can be achieved, resulting in a short current path; consequently, the internal losses are low [4].

**Figure 3:**
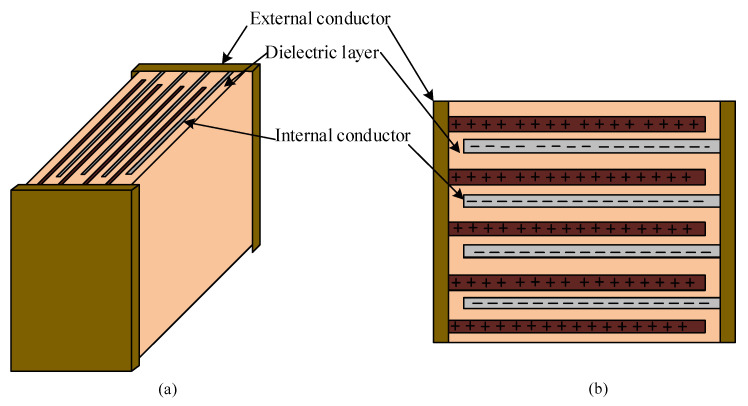
Construction of a high capacitance multilayer ceramic capacitor (MLC-Cap): (**a**) perspective view; (**b**) top view.

The configuration of an MLC-Cap, with the perspective and top views, is depicted in Figure 3. An MLC-Cap typically comprises several parallel internal conductor plates aligned within the dielectric layers, such that the distance between two neighboring conductors remains fixed. Hence, there are no gaps between the dielectric layers or the dielectric layer and the internal conductors. The capacitance value of the MLC-Cap can be controlled by regulating the length, space, or number of internal conductors [5].

**Figure 4:**
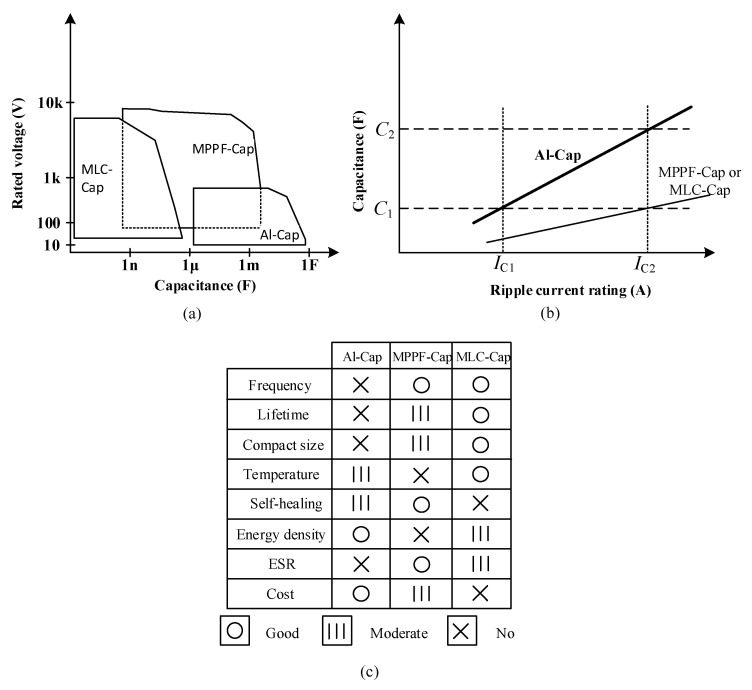
Comparisons of the three types of capacitors: (**a**) voltage range versus capacitance value; (**b**) required capacitance with respect to ripple current; (**c**) comparison based on various other parameters.

Figure 4 presents a comparison of the performances of three types of capacitors in terms of various parameters [6]. As shown in this figure, Al-Caps offer high energy density at low costs. However, they suffer from a high equivalent series resistance (*ESR*) and low ripple current. MLC-Caps feature a wide frequency range and high operating temperature; however, they are costly. On the contrary, MPPF-Caps offer a well-balanced performance, as compared with Al-Caps and MLC-Caps [6]. 

Applications of capacitors can be categorized into two types: high ripple current applications and low ripple current applications. Figure 4b shows the ripple current of the three types of capacitors. The ripple currents of these capacitors are proportional to their capacitance *C*. As shown in Figure 4, the required capacitance increases with the ripple current rating. In low ripple current applications, such as DC–DC converters, the capacitance values of *C_1_* are chosen for Al-Caps, MPPF-Caps, and MLC-Caps. In high ripple current applications, such as electric vehicles (EV), PV systems, and wind turbines, the required capacitance value for Al-Caps is *C_2_* due to the low ripple current rating, whereas the capacitance value for MPPF-Caps and MLC-Caps is *C_1_*. Failure of capacitors could occur due to various factors such as structure, wear out, operating temperatures, and electrical stress. Vaporization of the electrolyte is a major cause of failure in Al-Caps [7]. The dominant failure of MPPF-Caps is attributed to dielectric loss due to corrosion, which occurs at the inner and outer layers. When corrosion occurs, especially in the outer layer, the metal films are separated; this leads to a reduction in capacitance. Consequently, the capacitor reaches its end-of-life (EOL) [8]. Major failures of MLC-Caps are caused by factors such as insulation degeneration and flex cracking caused by the assembly process and improper handling [9]. Furthermore, the decrease in the thickness of the dielectric layer leads to an increase in leakage currents, which is a typical cause of insulation degradation. 

Al-Cap is the most popular capacitor type used in power converter systems because of its high volumetric efficiency, low cost and available in an enormous range of capacities and sizes [10]. When selecting capacitors there are two main properties that need to be considered: breakdown voltage and capacitance. The selection of initial capacitance value has to be taken into consideration of the normal and significant drop of capacitance in Al-Cap. If the over-voltages occur, the result is only a small decrease in capacitance of the MPPF-Cap and never a significant capacitance drop or a breakdown. Under the same situation, Al-Cap decreases a large amount of its capacitance, which results in an unbalanced voltage, and the capacitor can be in short-circuit mode in the worst case [11]. In addition, Al-Caps need connected in series and require voltage balancing resistors to maintain the required high level voltage in high power applications. This leads to a more complex bus structure and may cause additional damage when they fail. MPPF-Caps outperform Al-Caps in terms of *ESR*, self-healing capability, life expectancy, environmental performance, dc-blocking capability, ripple current capability, and reliability. However, the capacitance of the MPPF-Caps still cannot compete with Al-Caps, due to their relatively low volumetric efficiency and high cost [12]. Several comparisons were presented in [13,14,15] about the performance between Al-Cap and MPPF-Cap. It demonstrates that the MPPF-Cap can handle more ripple current due to the low *ESR* characteristic and their lifetime is about ten time longer than that of Al-Caps [16,17]. Furthermore, the cost of Al-Caps is also cheaper than that of MPFC-Caps. However, the operational lifetime of the Al-Caps is much shorter than that of MPPF-Caps, and so will most likely require replacing at least once or twice within the lifetime of the power converter. The MPFC-Cap has an operational lifetime more than ten times compared with Al-Caps. Then, the cost of replacement will outweigh the price difference between the different types of capacitors [18]. The other studies combine the Al-Caps and MPPF-Caps to significantly reduce heating of the electrolytic capacitors, which translates directly into much longer service life [19,20]. A hybrid bank combining the best features of Al- and MPPF-Caps has been developed in [19]. MPPF-Caps offer high current rating with relatively low capacitance density, while Al-Caps are the exact opposite with excellent capacitance density and low current capability. The addition of the MPPF-Cap extends the life of the capacitor bank by more than 10 years [19]. The hybrid capacitor bank maintains a longer lifetime compared with the full Al-Cap bank and a lower cost and volume compared with the full MPPF-Cap bank. However, the combined banks still have the following limitations: (1) the sizing criteria of the hybrid ratio is still an open question; (2) the hybrid ratio between two types of capacitor is mostly experience based [20]. Generally, MPPF-Caps have higher reliability and longer lifetime compared with Al-Caps. Hence, the major of health monitoring techniques are focused on Al-Caps.

**Figure 5:**
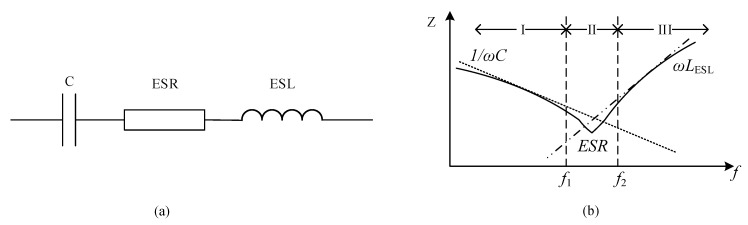
Equivalent circuit and dominant impedance of a capacitor: (**a**) equivalent circuit of capacitor; (**b**) dominant impedances in different regions of the capacitor.

A simplified circuit of a capacitor and a plot of the capacitor’s impedances corresponding to its frequency characteristics are presented in Figure 5a,b, respectively. The capacitor impedance is dominated by capacitance *C* in region I, *ESR* in region II, and the equivalent series inductance (*ESL*) in region III. In a power system, an individual capacitor or a bank of capacitors is frequently used for filtering, bypass, power decoupling, and energy buffering. Therefore, if a capacitor fails, the system could experience critical problems. In capacitor banks, the time to reach the EOL varies; hence, if one or more capacitors fail, the remaining capacitors undergo increased electrical stress, resulting in an increase in the rate of degradation. Hence, replacing all the capacitors in a bank when one or more capacitors fail is an effective measure to maintain the reliable operation of the system [21].

The use of *ESR* as an indicator for the health monitoring of capacitors has been reported by several studies [22,23,24,25,26,27,28,29,30,31,32,33,34,35,36,37,38,39]. However, these studies are only applicable for the condition monitoring of Al-Caps based on their high *ESR* characteristics. Conversely the *ESR* in MPPF-Caps is relatively low; therefore, using *ESR* as a health indicator could incur undesirable errors due to the high estimation errors in monitoring methods. These errors could lead to incorrect decisions regarding maintenance, thereby increasing expenditure. Various studies [40,41,42,43,44,45,46,47,48,49,50,51,52,53,54,55,56,57,58,59,60,61,62,63,64,65,66,67,68,69,70,71,72,73,74,75,76,77], which are discussed herein, have reported on the health monitoring of capacitors. Capacitance monitoring methods are capable of estimating the capacitance of both Al-Caps and MPPF-Caps; therefore, they would be more beneficial and suitable for different applications. This paper provides an overview of existing approaches for monitoring capacitors. The advantages and limitations of different monitoring methods are also identified and discussed herein.

## 2. Capacitor Condition Monitoring Methods 

**Figure 6:**
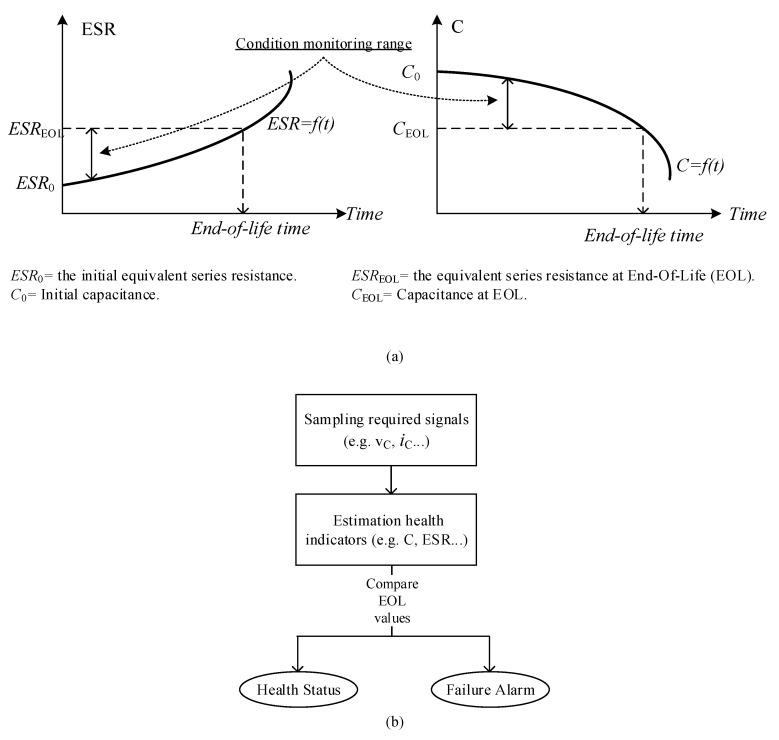
Indicators and general steps involved in condition monitoring of capacitors: (**a**) indication curves of capacitor degradation and (**b**) general steps of condition monitoring for capacitors.

Condition monitoring methods for both single capacitors and capacitor banks are based on the evaluation of the capacitance *C* and/or the *ESR*, which indicate the health status of a capacitor. The curves of capacitor degradation and the general scheme for condition monitoring of the capacitors are presented in Figure 6a,b [10]. The EOL of Al-Caps is realized at a capacitance reduction of 20% or twice that of the *ESR*. The EOL of MPPF-Caps is indicated by a capacitance reduction of 2–5%. 

### 2.1. ESR Estimation Methods 

**Figure 7:**
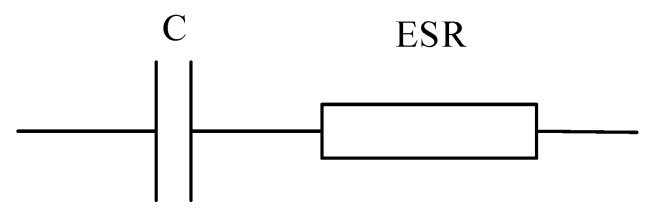
Simplified circuit of a capacitor.

The impedance of the simplified capacitor circuit in Figure 5(a) is expressed as

(1) $$Z_{cap}=\sqrt{ESR^{2}+{\left(ESL\cdot \omega -\frac{1}{C\omega }\right)}^{2}}$$

where ω=2πf is the frequency characteristic. If the capacitor functions in a low-band frequency (such as $f<f_{2}$; this is significantly common in practical capacitor applications), as shown in Figure 5b, then the *ESL* is sufficiently low to be neglected. Thus, the equivalent capacitor circuit in Figure 5a could be redrawn as presented in Figure 7. 

When f is located in region II, the capacitor impedance is dominated by the *ESR*. The *ESR* of a capacitor is calculated using the ratio of the capacitor’s voltage and the current for a certain range of frequency in region II, as in [23,33]:

(2) $$ESR=\frac{V_{C\_II}}{i_{C\_II}}$$

where $V_{C\_II}$ and $i_{C\_II}$ are the capacitor voltage and current in frequency region II.

Capacitor parameters and a temperature estimation method based on monitoring the *ESR* are proposed for a front-end rectifier-fed three-phase inverter in [30]. The *ESR* is estimated using the operating frequency components of the capacitor voltage and current, without neglecting the *ESL* (although it is commonly neglected in many capacitor monitoring techniques). The voltage drop on the *ESR* is extracted from the capacitor voltage by detecting the phase difference between the capacitor voltage and the *ESR* voltage, using band-pass filter (BPF). On obtaining the *ESR* drop voltage, the *ESR* of the capacitor can be estimated as [19]:

(3) $$ESR_{f}=\frac{V_{C\_fPEAK}\times cos\theta_{f}}{I_{C\_fPEAK}}$$

where $V_{C\_fPEAK}$, $I_{C\_fPEAK}$, and $\;\theta_{f}$ are the capacitor voltage, capacitor current, and phase difference, respectively; this phase difference is between the capacitor voltage and *ESR* voltage at the switching frequency. Considering the *ESL* in capacitor monitoring reduces the *ESR* estimation error and provides a more accurate result for predictive maintenance of the capacitor.

A monitoring technique for Al-Caps is proposed in [40]. The proposed technique offers a low cost and simplified method. In [40], the capacitor voltage $v_{C}$ is represented as a function of the input voltage $v_{in}$ [40]:

(4) $$\frac{v_{C}}{v_{in}}≅\frac{ESR}{R}+\frac{jZ_{cap}}{R}$$

where R is the input resistance. Thereafter, the capacitor parameters are estimated based on the Newton–Raphson algorithm. It is important to note that the input resistance value should be larger than the capacitor impedance due to the convergence issue. This technique provides simplification and high accuracy for capacitor parameter estimations [40].

### 2.2. Capacitance Estimation Methods 

Capacitance is estimated using the capacitor voltage $v_{C}$ and current $i_{C}$, as follows:

(5) $$i_{C}=C\frac{\Delta v_{C}}{dt}$$

(6) $$C=\frac{\int^{}i_{C}dt}{\Delta v_{C}}$$

where $\Delta v_{C}$ is the variation of capacitor voltage in the estimation interval.

The other technique in [56] injects a sinusoidal voltage signal to the output terminal of an isolating DC–DC converter. Subsequently, capacitance is estimated using the impedance of the capacitor, which is determined as [56]:

(7) $$X_{C}=\frac{1}{2\pi f_{m}C}$$

where $f_{m}$ is the frequency range dominated by the capacitor’s impedance. This technique does not require any additional current sensors or hardware modifications. However, injecting voltage signal may cause undesired disturbances in the system.

**Figure 8:**
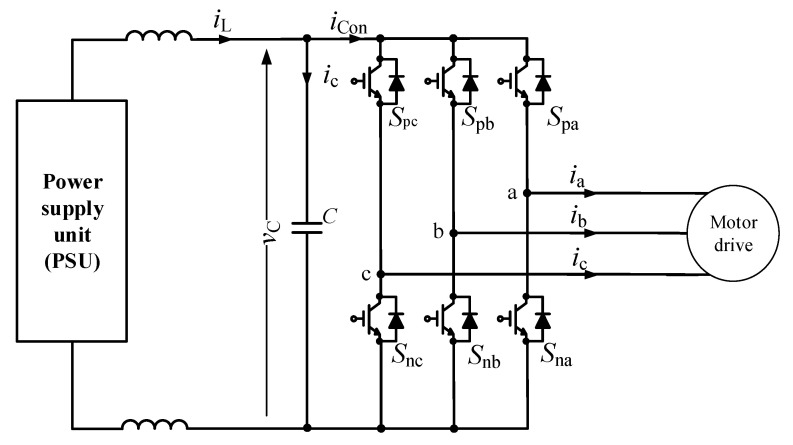
Motor drive topology.

Online capacitance monitoring of a capacitor is presented in [57]. A motor drive topology is demonstrated in Figure 8. When the switches change their ON-OFF status, ripple voltages are caused on the capacitor. These ripples are isolated from the source by the inductors. The capacitance of the capacitor is calculated using Equation (6). To avoid the presence of the capacitor current sensor, the capacitor current can be evaluated using the difference between the inductor current $i_{L}$ and the converter current $i_{Con}$. In a similar manner, the converter current could be calculated using the relationships between the ON-OFF statuses of the switches and the converter phase currents ($i_{a},\;i_{b},\;i_{c}$). The advantage of this method is that additional component or signal injections are not required, which results in a low cost and small size solution for capacitor monitoring. However, the reliability of this method depends on the accuracies of the sensors due to the small ripple voltage of the capacitor, which is approximately 1% of the capacitor voltage. Thus, appropriate design of the shielding and ground wiring layout is essential to attenuate noises [57].

Several advanced algorithms, such as the Adaptive Neuro-Fuzzy Inference System (ANFIS) and the Artificial Neural Network (ANN) algorithms, have been adapted to monitor the health status of capacitors [58,59,60,61,62]. The ANFIS detects aging faults of the capacitors in the converter based on the aging relationships among the estimated EOL and the actual capacitor voltages by using curve fitting techniques. Utilizing the input data created at the normal and aging fault conditions of the capacitors, the ANFIS is capable of monitoring the health of capacitors [58]. The ANN algorithm structure comprises three layers: the data input layer, processing hidden layer, and capacitor health indicator output layer. The input data of the ANN, such as input current and voltage, capacitor voltage, and output current and voltage, are stored in the input layer. The hidden layer transforms the input data into training function data suitable for the output layer; thereafter, the target data are modified as health indicators, such as the capacitance of the capacitor. Advanced algorithms have achieved high accuracy solutions for health monitoring of capacitors, thereby eliminating the need for additional hardware such as capacitor current sensors; therefore, these algorithms are preferable for practical applications [62]. However, powerful processors are required to execute the heavy computations and complex algorithms.

### 2.3. ESR and Capacitance Estimation Methods 

Adopting the same monitoring circuit as the method in [41] uses the discrete Fourier transform (DFT) algorithm to estimate both the *ESR* and the capacitance of a capacitor. Both the phase and modulus of the fundamental voltage and current of a capacitor are computed using the DFT algorithm, as follows [41]:

(8) $$f_{1st}≅\overset{NPP}{\underset{n=1}{\sum }}\frac{f\left(n\right)\times e^{\omega_{0}nj}}{0.5\;NPP}$$

where $f_{1st}$, NPP, and $\omega_{0}$ are the fundamental harmonic of the waveform f(n), total number of points per period, and radian frequency, respectively. Then, the real and imaginary capacitor impedances are estimated as [41]:

(9) $$ESR≅\frac{\left|v_{C}\right|}{\left|i_{C}\right|}\cos\left(|\underline{v_{C}}-|\underline{i_{C}}\right)$$

(10) $$X_{C}≅\frac{\left|v_{C}\right|}{\left|i_{C}\right|}\sin\left(|\underline{v_{C}}-|\underline{i_{C}}\right)$$

where $X_{C}$, $\left|v_{C}\right|,$ and $\left|i_{C}\right|$, $|\underline{v_{C}}$ and $|\underline{i_{C}}$ are the capacitance impedance, modulus of capacitor voltage and current, the phase angle of capacitor voltage and current, respectively. Interestingly, the accuracy of *ESR* estimation increases at higher frequency such as 1 kHz, whereas the accuracy of capacitance estimation increases at lower frequency such as 120 Hz. The techniques in [40,41] yield high accuracy in monitoring the health of a capacitor, while being cheap and simple [41]. However, they are offline methods that require isolating the capacitor from the operation mode.

The online scheme estimates capacitor parameters using sliding mode differentiators and recursive algorithms, as reported in [47,48]. The capacitor voltage and current can be affected by noise; therefore, the sliding mode differentiator is used to provide satisfactory derivatives. Thereafter, the recursive least square algorithm is adapted to estimate the capacitor’s parameters. The proposed approaches only employ a mathematical model for the capacitor rather than a model of the system to which the capacitor is connected. The primary advantage of these approaches is that they are capable of achieving highly accurate estimations of both the *ESR* and capacitance, even under noisy conditions [47]. However, the requirement of the additional capacitor current sensor results in additional costs of the converter.

The other monitoring technique proposed in [49] estimates *ESR* and capacitance by using large-signal load transient trajectories at low sampling frequencies. Regarding the sampling waveforms, the sampling frequency of the large-signal transient voltage need not be higher than that of the steady state voltage in order to achieve the same quality of sampling waveforms [49]. Hence, instead of sampling the steady voltage and current, the large-signal transient voltage and current are sampled and utilized to estimate the capacitor parameters, without requiring a duty cycle signal as in [43,44]. However, this technique is only applicable to transient states with large signals. Moreover, modifying converter control to provide large-signal transient trajectories could cause unstable issues with the converter operation. 

The technique in [65] applies methods similar to that in [30], without requiring an analog filter to extract the high-frequency capacitor voltage and current components using the Goertzel algorithm. There are three stages in the proposed scheme: the input sampling signal, estimation processing stage, and health indicator output stage. The input stage is the sensing signal for ambient temperature, capacitor current, and voltage. The estimation stage is the most important stage because it processes the input signals to estimate the *ESR*, capacitance, and core temperature of the capacitor. The input signals, after passing through the analog-to-digital (AD) converter, are extracted using the Goertzel algorithm to obtain the required current and voltage. Thereafter, the *ESR* and capacitance are estimated, and the estimated capacitance value is utilized to calculate core temperature. The present health status of the capacitor is computed and sent to the output stage as an indicator of the capacitor’s health. This method utilizes the Goertzel algorithm to eliminate the use of analog filters, thereby improving the computation cost ratio [65]. However, a capacitor current sensor is required in both the monitoring schemes [30,65], which is not attractive for practical applications.

### 2.4. Other Health Monitoring Methods

Metallized polypropylene films are used in capacitors owing to their self-healing abilities. Whenever self-healing process occurs, the local defects in the MPPF-Cap are eliminated, resulting in a small loss of capacitance. A monitoring technique for MPPF-Caps is proposed in [42]. The decrease in capacitance leads to an increase in nonlinearities of the current and voltage characteristics. The techniques in [42] are based on the nonlinearity characteristic measurement for monitoring capacitor degradation during thermal aging. If the nonlinearity characteristic has the form of an even or odd parabola, the odd nonlinearity rate could be measured using the highest amplitude of the even or the odd harmonic component, respectively. The thin film in MPPF-Caps is typically composed of aluminum [42]; therefore, the third harmonic is measured as [42]:

(11) $$THI=20\times \log\frac{U_{3}}{U_{1}^{n}}$$

where THI, $U_{3}$, $U_{1}$, n are the third harmonic index, the third harmonic measured voltage, the fundamental harmonic measured voltage, and a parameter equal to 3, respectively. It should be noted that the changes for higher value capacitors are greater, whereas the changes for the same type of capacitor can be different and depend on each capacitor’s quality. The proposed technique effectively monitors the health status of MPPF-Caps by measuring the change in nonlinearity characteristics. However, this technique requires a significant amount of time to measure the nonlinearity changes.

## 3. Condition Monitoring Methods for Capacitors in Power Converters

### 3.1. ESR Estimation Methods

**Figure 9:**
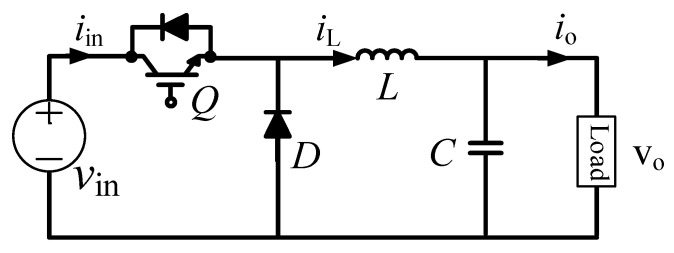
Buck converter.

**Figure 10:**
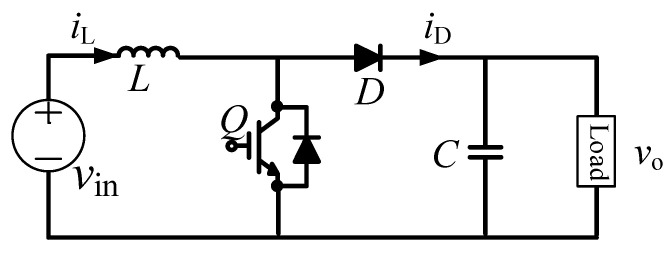
Boost converter.

Al-Caps are widely used in DC–DC converter systems, which fail more frequently when compared with other electronic devices. During operation, the performance of the Al-Cap undergoes continuous degradation until the capacitor reaches its EOL and the system fails. Therefore, calculating the *ESR* and capacitance *C* of a capacitor is essential for analyzing the health status of capacitors in a DC–DC buck converter, as shown in Figure 9. Unlike buck converters, which provide an output voltage lower than the input voltage, the output voltage of a boost converter is higher than the input voltage. A general circuit for a boost converter is presented in Figure 10.

Another monitoring technique proposed in [31] involved the use of tunneling magneto-resistive (TMR) sensors in order to measure the input capacitor and inductor currents of a PV boost converter system; the other measurements are captured using existed sensors. Based on the relationships among inductor current, output and input voltages, output capacitor voltage, and current based on switching operations, the *ESR*s of the input and output capacitors could be estimated in both the continuous conduction mode (CCM) and the discontinuous conduction mode (DCM) [31].

An online *ESR* estimation scheme is proposed in [38,39] for buck and boost converters, under both CCM and DCM. The method only measures inductor current ripple and capacitor voltage ripple for *ESR* estimation; therefore, additional sensors are not required. This results in lower costs and easier implementation [38]. In practical applications, measurements of the inductor current ripple and capacitor voltage ripple typically contain undesired noise, such as white and random noises [39]. Hence, the measured signal is preprocessed prior to the estimation of *ESR*. Wavelet transformed de-noising (WTD) is selected for the preprocessed measured signal. This method is capable of estimating the *ESR* under different loads such as resistive, inductive, and converter loads. 

### 3.2. Capacitance Estimation Methods

In AC–AC converters, capacitors are frequently utilized in the DC-link. Depending on the application, one or several capacitors could be connected in series or parallel.

**Figure 11:**
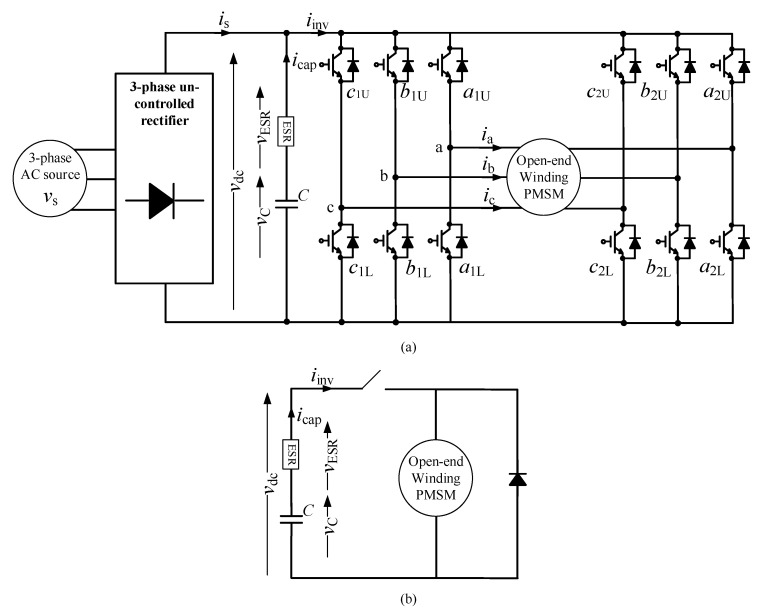
Circuit diagram of the permanent magnet synchronous machine (PMSM): (**a**) circuit diagram; (**b**) equivalent circuit during the discharge period when the machine stops.

A monitoring algorithm for capacitance estimation by injecting a zero-sequence current for an open-end winding permanent magnet synchronous machine (PMSM) is proposed in [63]. This monitoring algorithm only uses the measurements in the discharging period. The circuit diagram and equivalent circuit of the discharging period are shown in Figure 11. The discharging period is identified by the derivative of the DC-link capacitor $v_{dc}^{\prime }$. At the beginning of the discharging process, the DC-link capacitor begins to discharge through the inverter when $v_{dc}^{\prime }$ < 0 and stops charging when $v_{dc}^{\prime }$ > 0. A zero-sequence current is injected to control the discharging capacitor current, when the machine is in the standstill condition. Thereafter, the discharging capacitor current is estimated by separately analyzing each individual phase, and the result is obtained using the superposition theory, as follows [63]:

(12) $$i_{cap}=\left(da_{1U}-da_{2U}\right)\times i_{a}+\left(db_{1U}-db_{2U}\right)\times i_{b}+\left(dc_{1U}-dc_{2U}\right)\times i_{c}$$

where $i_{cap}$, $i_{a}$, $i_{b}$, $i_{c}$, $da_{1U},\;\;da_{2U}$, $db_{1U}$, $d2_{2U}$, $dc_{1U}$, $\mathrm{and}\;dc_{2U}$ are the capacitor current, *a*-phase current, *b*-phase current, *c*-phase current, and duty cycles of switches $a_{1U}$, $a_{2U}$, $b_{1U}$, $b_{2U}$, $c_{1U}$, $\mathrm{and}\;c_{2U}$, respectively. Capacitance is estimated using the recursive least square method and is expressed as [63]:

(13) $$\hat{C}\left(n+1\right)=\hat{C}\left(n\right)+\gamma \left(n\right)LBF\left(v_{dc}^{\prime }\right)\times \left[i_{cap}\left(n\right)-\hat{C}\left(n\right)LBF\left(v_{dc}^{\prime }\right)\right]$$

where γ(n) and $\mathrm{LBF}\left(v_{\mathrm{dc}}^{\prime }\right)$ are the constant adjustable gain and the derivative of $v_{\mathrm{dc}}$ after passing through the low pass filter (LPF). Simplification of the control system, achieved by the current injection technique, is a primary advantage of this method; however, this method is only effective when the machine is in the standstill mode [63].

In [68], a noninvasive technique for capacitance estimation is developed. Only the input voltage $V_{\mathrm{in}}$, current $i_{\mathrm{in}}$, and output current $i_{\mathrm{out}}$ are required, sensed, and sampled in order to generate the pulse width modulation (PWM) signal m. Thereafter, the capacitor current is evaluated to detect the zero crossing points. Thus, the accumulation of charge on the capacitor between the interval of two continuous zero crossing points could be evaluated and combined with the switching period $T_{s}$, in order to determine the net current charge on the capacitor. Similarly, the net voltage change is also estimated, and the capacitance is computed as a ratio of the net current charge and the net voltage change. An initial value of the capacitance is compared with the estimated capacitance to indicate the health status of the capacitor. Additional sensors are not required for health monitoring when using this technique [68]. However, utilizing the second harmonic of capacitor voltage is not suitable for systems without frequency oscillations, such as DC–DC converters [71].

**Figure 12:**
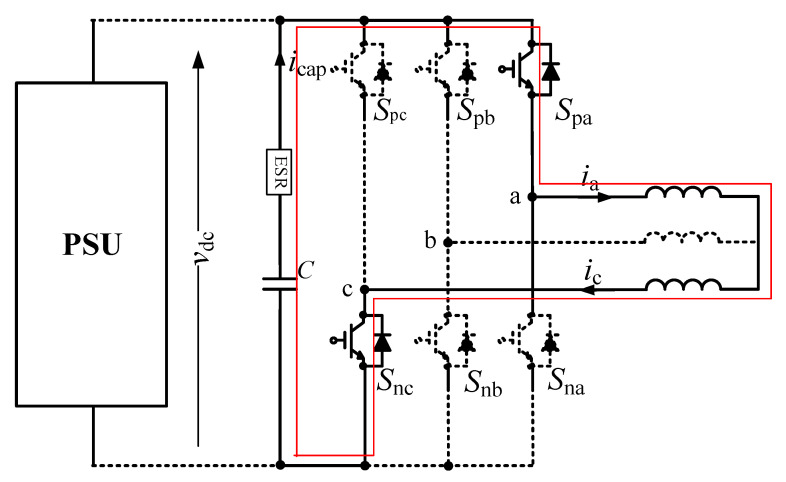
Form of *LC* resonance in the voltage source inverter (VSI) system.

A forming *LC* resonance between the DC-link capacitor and the inductance for estimating the capacitor’s condition when the converter is stopped is introduced in [70]. By switching insulated gate bipolar transistors (IGBTs), the capacitor discharges through the phases of the machine (e.g., *A* and *C*), forming an *LC* resonance network, as shown in Figure 12. This results in a decrease in the capacitor voltage and an increase in the capacitor current. After the energy in the capacitor is completely discharged, the capacitor voltage equals zero, and the resonance current is captured for data processing and slowly drops to zero. The capacitance is evaluated as a multivariate nonlinear regression (MNR) model [70]:

(14) $$C=\frac{\beta_{1}\times \beta_{3}}{V_{0}\left(\beta_{2}^{2}+\beta_{3}^{2}\right)}$$

where $\beta_{1}$, $\beta_{2}$, and $\beta_{3}$ are the estimated variables of the resonance current obtained using the iterative least square (ILS) algorithm. The presented method enables high accuracy estimations for capacitance monitoring, without requiring additional hardware or signal injections [70]. However, this approach is only effective when the converter is in the standstill mode, and the formation of the *LC* resonance is critical to achieve an accurate estimation.

**Figure 13:**
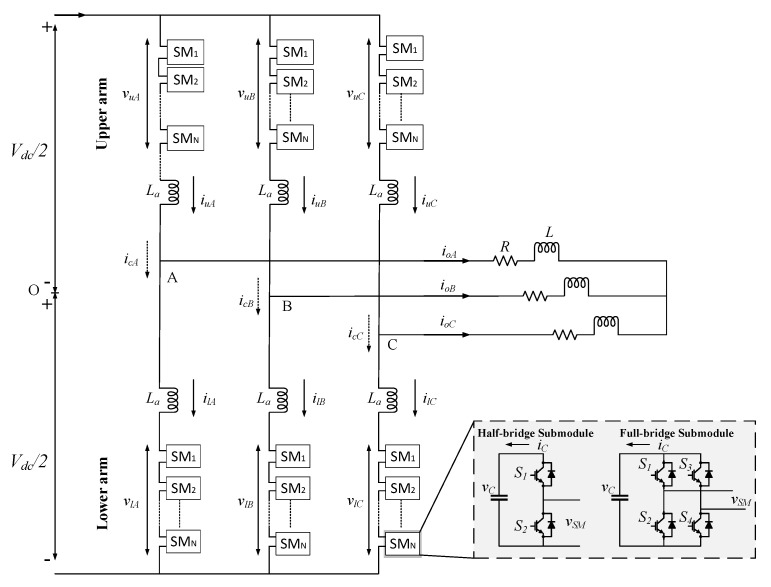
Topology of modular multilevel converter (MMC) with half-bridge and full-bridge configurations.

Multilevel modular converter (MMC) is an emerging topology for high-voltage and high-power applications that has attracted significant interest owing to its advantages such as modularity, scalability, low manufacturing difficulty, and high efficiency. The MMC comprises several submodules (SMs), which could include half-bridge (HB) converters or full-bridge (FB) converters, as shown in Figure 13. Each submodule (SM) has its own capacitor for filtering and energy storage; therefore, due to the numerous capacitors used in an MMC, ensuring reliability in this system is critical. Therefore, health monitoring of these capacitors is required to maintain a stable and safe operation of MMCs.

In [72,73], reference submodule (RSM)-based capacitance estimations for MMCs with FB and HB SMs are proposed, respectively. An SM is selected as an RSM, which features the highest capacitance among monitoring SMs (${\mathrm{SM}}_{1}-{\mathrm{SM}}_{N}$) in the arm. The remaining SMs are sorted according to the increasing order of capacitor voltages. Subsequently, the capacitance of each SM is estimated based on the difference in capacitor voltages of the RSM and the monitoring SM [73]:

(15) $$C_{i}=C_{RSM}\frac{\Delta v_{RSM}}{\Delta v_{i}}$$

where $\Delta v_{RSM}$ and $\Delta v_{i}$ are the ripple values of the RSM voltage and the monitoring SM voltage, respectively. The RSM method provides an effective solution for capacitance monitoring of a capacitor, without requiring additional hardware [73]. The reliability of the RSM method is mainly based on the appropriate selection of an RSM; thus, an accurate selection process is necessary.

**Figure 14:**
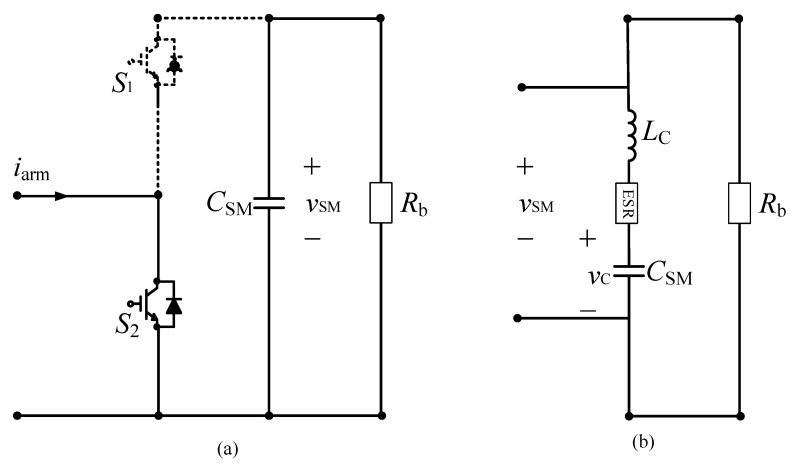
Operation circuits of an submodule (SM) (**a**) resistor-capacitor (*RC*) discharging circuit; (**b**) equivalent circuit in discharging mode.

In [75,76], the SM from the operating MMC was isolated and the curve of the capacitor voltage in the discharging process was utilized to obtain the health condition of a capacitor. During the discharging process shown in Figure 14, the state of switch $S_{2}$ is ON, whereas that of switch $S_{1}$ is OFF. Thus, the capacitor only discharges through the bleeding resistor $R_{b}$, which absorbs the energy of the capacitor when the SM is not in operation. The health condition of the capacitor could be monitored through the decrease in discharging time, as follows [76]:

(16) $$\frac{\partial t_{0.368}}{\partial ESR_{i}}=C$$

where $t_{0.368}$ is the discharge time when the capacitor voltage $v_{i}$ decreases from the initial value $v_{i\_0}$ to $0.368v_{i}$. The SM is re-connected to the MMC when the voltage of the capacitor is lower than $0.368v_{i}$. The health of capacitors is judged using the same process for the remainder of the SMs in the MMC. This method monitors the capacitor of the SM without necessitating heavy computation or requiring the arm current or present switching state. However, isolating an SM from an operating MMC could lead to an increase in the electrical stress acting on the remaining SMs; it can also lead to negative effects on the output voltage and current in the absence of redundant SMs or if the number of SMs is insufficient [76].

### 3.3. ESR and Capacitance Estimation Methods

In [43,44,45], online monitoring methods for the buck converter are proposed. The primary advantage of these methods is that, instead of measuring both current and voltage capacitors, only two values of the capacitor voltage at two specific moments within a switching period need to be measured. By analyzing the capacitor ripple voltage, the calculation models of both *ESR* and *C* in [43,44] are constructed as

(17) $$ESR=\frac{2Lf_{s}\left\{\left[v_{o}\left(0\right)-V_{o}\right]+\frac{2\left(2D-1\right)}{\left(2-D\right)}\times \left[v_{o}\left(\frac{DT_{s}}{2}-V_{o}\right)\right]\right\}}{V_{o}\left(D-1\right)}$$

(18) $$C=\frac{V_{o}\left(2-D\right)\left(1-D\right)}{24Lf_{s}^{2}\left[V_{o}-v_{o}\left(\frac{DT_{s}}{2}\right)\right]}$$

where $L,\;f_{s},\;V_{o},\;D,\;T_{s}$ are the inductance, switching frequency, output voltage, duty cycle, and switching cycle, respectively.

In [43], where the trigger circuit generates trigger signals at 0 and $DT_{s}/2$ moments using the PWM signal from the control unit. The AD converter in the micro-control unit (MCU) analyzes the ripple in the output voltage after extracting and amplifying it by using an isolated amplifier; this extraction and amplification is conducted when the trigger signals first appear, while the pulse capture is active. Combining the switching frequency and duty cycle signals obtained from the trigger circuit, the calculation models of the *ESR* and *C* are obtained, and the final result is displayed in real time. These methods offer a health monitoring solution without requiring current sensors. However, these methods are only effective in the CCM of buck converters [43].

Several existing methods for buck converters [38,43,44] are capable of applying boost converters [39,51,52]. Monitoring techniques for the fly-back converter and the isolating DC-DC converter are proposed in [54,55,56]. The approaches in [43,44] are also adapted for fly-back converters in [54,55]. The difference between this methods and previous methods is an increase in the number of sampling points and the application of the least square algorithm for the measured noise reduction. The advantages and disadvantages are similar to those of previous techniques, i.e., although the monitoring scheme only requires voltage signal measurements, it is effective in CCMs [54] or DCMs [55].

A state observer method for monitoring the health condition of capacitors is proposed in [46]. Voltage injection, which is a low-frequency signal, such as a 100 Hz square wave, is used to avoid the need for additional hardware. Due to the injected voltage signal, the variation of the capacitor voltage is larger; hence, it can be sampled by a normal voltage sensor and processing devices. The state observer is used to estimate the capacitor voltage. Thereafter, the *ESR* and *C* can be obtained and adjusted according to the difference between the estimated voltage and the actual voltage value; this is continued until the difference reaches zero. This method achieves the high accuracy for the estimation of capacitor health status, without requiring additional hardware [46]. However, injecting a low-frequency voltage signal results in an increase in voltage ripple, thereby necessitating a larger capacitor; this increases the size and cost of the converter.

A simplified method to detect changes in the *ESR* and the capacitance of a capacitor is proposed in [50]. The capacitor’s voltage and current are measured and passed through the BPF with a frequency range in the region of dominance of the *ESR* or capacitance. The output signals of the BPFs are continuously subjected to root mean square (rms) calculations. Thereafter, automatic gain controllers are used to obtain the ratio of the capacitor ripple voltage and the capacitor current, i.e., the impedance of a capacitor, which is approximately equal to the *ESR* or capacitance impedance depending on the frequency range of the BPF. The *ESR* and the capacitance impedance $X_{C}$ of the capacitor are estimated using Equations (2) and (7). Thus, this scheme achieves highly accurate estimations using a noncomplex algorithm [50]. However, this method requires additional hardware, such as capacitor current sensors, which increases the size and cost of the converter.

The technique in [53] considers the voltage drop on the capacitor required to improve the *ESR* and capacitance estimation accuracy. The capacitor impedance is dominated by the *ESR* when the operating frequency is tens to hundreds of kilohertz, and the *ESR* is typically estimated using Equation (2). However, the capacitor’s dropped voltage was not considered in previous approaches [23,32,33], which could lead to estimation errors of the *ESR*. The proposed technique considers the capacitor’s dropped voltage in order to estimate the *ESR* and capacitance. However, the presence of TMR sensors results in an increase in size, cost, and power loss of the system.

A condition monitoring technique for DC-link capacitors in medium- and high-power AC–DC–AC PWM converters based on the designed variable electrical network (VEN) is proposed in [64]. Several capacitors are connected in series as a capacitor bank to maintain the required voltage of the DC-link. The balance resistors $R_{j}$ are connected in parallel with the capacitors in order to sustain equal voltages on each capacitor. The single VEN consist of two brands $X_{1}$ and $X_{2}$; each brand comprises a switch and a resistor. The estimation of capacitor parameters is conducted during the shutdown period. The MCU controls the VENs and calculates the parameters of the capacitor. During the shutdown period, the DC-link capacitors are isolated from the load as well as the source and then discharged through the VENs. The discharging period is divided into three intervals ($T_{0}$,$T_{1}$,$T_{2}$). In the first interval, both switches $G_{1}$ and $G_{2}$ are turned off; thus, the capacitors only discharge through the balance resistors. In the second interval, the balance resistors and brand $X_{1}$ are discharged when switch $G_{1}$ is turned ON. During the third interval, the balance resistors and brand $X_{2}$ are discharged when the state of switch $G_{2}$ is ON, and the state of switch $G_{1}$ is OFF. Each discharged interval has a different time constant ($\tau_{0}$,$\tau_{1}$,$\tau_{2}$), and the time constants ($\tau_{1}$,$\tau_{2}$) could be estimated by using the discharged voltage in the second and the third intervals. Thus, the capacitance and *ESR* can be estimated as [64]:

(19) $$C=\frac{\tau_{1}}{\left(R_{j}||R_{X1}\right)}$$

(20) $$ESR=\frac{\tau_{2}}{C}-R_{X2}$$

where $R_{X1}$ and $R_{X2}$ are the resistances of brands *X*_1_ and *X*_2_, respectively. The proposed technique monitors the health of capacitors without requiring capacitor current information, which prevents the noise from measurements or specific requirements such as the bandwidth and frequency response speed. However, the technique is only effective in the shutdown period [64], and the presence of VENs increases the weight, volume, and cost of the converter system.

A condition monitoring method for single-phase solar inverters is proposed in [66,67]. Various order harmonics (only odd orders, from the third to eleventh orders), which account for just 4% of the total rated grid current of the converter, are injected, and the samples of the capacitor voltage and output current are captured. Subsequently, the capacitor current is estimated using the relationship between the output current and the converter switching states. Thereafter, the rms capacitor impedance is computed using the capacitor current and voltage after passing through the BPF. Utilizing the least mean square (LMS) algorithm to estimate the *ESR* and the capacitance of the capacitor and by comparing this with the initial capacitor values at the current operating temperature, the health status of the system can be deduced. As an advantage, this monitoring method only requires existing sensors and also accounts for the variation temperature effect. However, this approach is only applicable at night when the solar panels do not generate a voltage; it also requires a harmonic current injection [67].

**Figure 15:**
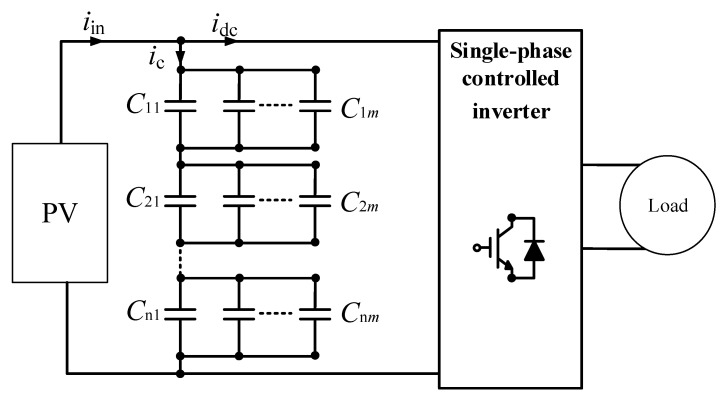
Single-phase photovoltaic (PV) system with capacitor bank.

The primary function of an inverter is to convert DC signals to a single-phase or three-phase AC signal with variable magnitude and frequency and vice versa, in order to function as a rectifier. A single capacitor or a bank of capacitors are frequently used as the filters at the DC side. Hence, the condition monitoring of capacitors is essential in order to maintain high-performance converters. When one or several capacitor banks are utilized, monitoring methods using the capacitor’s current sensor to estimate the health of individual capacitors cannot be employed due to the increase in the required current sensors, which leads to an increase in weight, volume, and cost of the system. A condition monitoring technique for individual capacitors in a bank is proposed in [69]. The circuit configuration is presented in Figure 15. As shown in Figure 15, the capacitor bank is placed between the solar panel and the converter in order to attenuate ripple current, which is caused by the switching operation of the converter and the oscillations of the AC source. This monitoring scheme consists of various stages: (1) first-start calibration of the capacitor; (2) estimation of the capacitor’s current; (3) estimation of the capacitor’s core temperature; (4) estimation of the capacitor’s degradation; (5) estimation of capacitor’s bank parameters; and (6) capacitor model updating. The initial calibration stage is used to calibrate the initial *ESR* and the capacitance values at the first-time start of the converter. The second stage estimates the capacitor current based on the relationship between the input and output currents and the switching states of the converter. Thereafter, the capacitor’s core temperature is evaluated using heat flow equations in the third stage. Subsequently, the degradation of individual capacitors is estimated using the physics of failure (PoF)-based model. Thus, the parameters of a capacitor at the [*n*+1]th instant are expressed as [69]:

(21) $$C_{i}\left[n+1\right]=C_{T}\left[n\right]\times C_{0i}\times \left(1-\alpha_{i}\left[n\right]\right)$$

(22) $$ESR_{i}\left[n+1\right]=ESR_{T}\left[n\right]\times ESR_{0i}\times e^{\beta_{i}\left[n\right]}$$

where $C_{0i}$ and $ESR_{0i}$, $C_{T}$ and $ESR_{T}$, $\mathrm{and}\;\alpha_{i}$ and $\beta_{i}$ are the initial capacitance and *ESR* values of the *i*th capacitor at ambient temperature, the capacitance and *ESR* values of the *i*th capacitor at core temperature, and the temperature- and time-dependent variables, respectively. In the absence of a failed capacitor, the next stage is executed. Based on the estimated capacitances and *ESR*s of individual capacitors, the capacitance and *ESR* of the capacitor bank are calculated. In the final stage, the bank parameters are updated and compared with actual parameters. Based on the error between the estimated and actual parameters, the degradation coefficients are updated using the extended Kalman filter (EKF) algorithm. This technique does not require additional sensors for each capacitor in the bank; the required measurements can be realized using existing sensors. However, the measurements and calibrations of individual capacitors in the first stage could be challenging due to the large number of capacitors [69].

The sorting technique is also applied in [74] to sort the SM, which features the highest *ESR* or the lowest capacitance in each arm of the MMC. The sorting process is based on the relationship between the energy and *ESR* and the current and capacitance of each SM; it is expressed as [74]:

(23) $$K_{i}=\frac{2\pi ESR_{i}}{\omega_{1}}\left(1+\frac{2U_{i\_2f}^{2}}{U_{i\_1f}}\right)$$

(24) $$U_{i\_1f}=\frac{I_{i\_1f}}{\omega_{1}C_{i}}$$

where $\omega_{1}$, $I_{i\_1f}$, $U_{i\_1f}$, $\mathrm{and}\;U_{i\_2f}$ are the fundamental angular frequency, amplitude of the fundamental capacitor current component, and amplitudes of the fundamental and second-order capacitor voltage components, respectively. $K_{i}$ and $I_{i\_1f}$ are proportional to the *ESR* and capacitance. The SM, which has the biggest $K_{i}$ or the lowest $I_{i\_1f}$ is first sorted, and the parameters are estimated based on the monitoring techniques in [41,77]. Using this sorting process, the health monitoring of the MMC equipped with numerous capacitors is simplified. However, estimating the capacitance by injecting an AC current into the circulating current loop could cause undesired disturbances.

## 4. Conclusions

This research provides a brief overview on the structure, characteristic, specifications, lifetime, failure mechanisms, and comparison of three-type capacitors and a detailed overview of health monitoring methods for different capacitor types used in power electronic circuits. Different techniques that have been employed for the estimation of *ESR* and capacitance are also reviewed and discussed herein. Based on the reviewed techniques, the following key features are noted:

(1) Among three types of capacitors, MPPF-Caps offer a well-balanced performance compared with Al-Caps and MLC-Caps. However, Al-Caps is the most popular capacitor type used in the power converter systems due to its high energy density and low cost.
(2) Most power converter systems use Al-Caps and the *ESR* is the most popular health indicator for Al-Caps. Both *ESR* and capacitance can indicate the capacitor health status, and combining *ESR* and capacitance estimations provides capacitor monitoring techniques of higher accuracy. Regarding MPPF-Caps, the estimation of *ESR* cannot be used for health monitoring due to the fact that the *ESR* of MPPF-Caps is very small. Thus, the capacitance is a more preferred indicator than *ESR* for MPPF-Caps.
(3) Online techniques are utilized more frequently than offline techniques. However, the capacitor degradation is usually considered slow and the offline techniques offer greater simplicity and accuracy in monitoring the health of capacitors.
(4) Methods employing the capacitor’s current sensor are not preferred due to the additional requirements of hardware, which result in an increase in the weight, volume, and cost of the system.
(5) Advanced algorithms have emerged as promising techniques that do not require additional hardware; these algorithms provide highly accurate solutions for monitoring the health of capacitors. They are also reliable and offer high scalability for large-scale systems.

We suggest that future research on the condition monitoring of capacitors should focus on the following notions:

(1) Advanced algorithms with no additional hardware are very attractive when they can be applied to various types of power converter structures by upgrading the estimation algorithms.
(2) Other monitoring techniques need to achieve a better integration of additional hardware and a more cost-effective design to provide reliable and cost-effective monitoring solutions.
